# Forskolin Regulates L-Type Calcium Channel through Interaction between Actinin 4 and β_3_ Subunit in Osteoblasts

**DOI:** 10.1371/journal.pone.0124274

**Published:** 2015-04-22

**Authors:** Xuemei Zhang, Fangping Li, Lin Guo, Hongya Hei, Lulu Tian, Wen Peng, Hui Cai

**Affiliations:** 1 Department of Pharmacology, School of Pharmacy, Fudan University, 826 Zhangheng Road, Pudong New District, Shanghai, 201203, China; 2 Department of Pharmacy, Jing’an District Center Hospital of Shanghai (Huashan Hospital, Fudan University, Jing’an Branch), 259 Xikang Road, Shanghai, 200040, China; 3 Department of Nephrology, Putuo Hospital, Shanghai University of Traditional Chinese Medicine,164 Lanxi Road, Shanghai, 200062, PR China; 4 Renal Division, Department of Medicine, Emory University School of Medicine, Atlanta, GA, 30322, United States of America; 5 Renal Section, Atlanta Veteran Administration Medical Center, Decatur, GA, 30033, United States of America; Vanderbilt University Medical Center, UNITED STATES

## Abstract

Voltage-dependent L-type calcium channels that permit cellular calcium influx are essential in calcium-mediated modulation of cellular signaling. Although the regulation of voltage-dependent L-type calcium channels is linked to many factors including cAMP-dependent protein kinase A (PKA) activity and actin cytoskeleton, little is known about the detailed mechanisms underlying the regulation in osteoblasts. Our present study investigated the modulation of L-type calcium channel activities through the effects of forskolin on actin reorganization and on its functional interaction with actin binding protein actinin 4. The results showed that forskolin did not significantly affect the trafficking of pore forming α_1c_ subunit and its interaction with actin binding protein actinin 4, whereas it significantly increased the expression of β_3_ subunit and its interaction with actinin 4 in osteoblast cells as assessed by co-immunoprecipitation, pull-down assay, and immunostaining. Further mapping showed that the ABD and EF domains of actinin 4 were interaction sites. This interaction is independent of PKA phosphorylation. Knockdown of actinin 4 significantly decreased the activities of L-type calcium channels. Our study revealed a new aspect of the mechanisms by which the forskolin activation of adenylyl cyclase - cAMP cascade regulates the L-type calcium channel in osteoblast cells, besides the PKA mediated phosphorylation of the channel subunits. These data provide insight into the important role of interconnection among adenylyl cyclase, cAMP, PKA, the actin cytoskeleton, and the channel proteins in the regulation of voltage-dependent L-type calcium channels in osteoblast cells.

## Introduction

Voltage-dependent L-type calcium channels facilitate the converting of a voltage signal into an ionic signal with increase of intracellular calcium. They thus, are essential for the regulation of many calcium-dependent cellular processes. Voltage-dependent L-type calcium channels are present in osteoblasts [1] and osteoclast [2] cells, and the activation of these calcium channels in osteoblasts correlates with increased bone density [1] and decreased bone resorption [3]. Previous studies indicate that intracellular calcium plays a fundamental role in bone deposition through mechanotransduction pathways. Calcium channel activities influence mechanical load-induced bone formation. Application of cyclic strain to the substratum resulted in increased incorporation of calcium in osteoblast Ros 17/2.8 cell cultures, and the response is diminished in the presence of verapamil, a blocker of voltage-dependent calcium channels [4]. When treating rats with verapamil and nifedipine, another L-type calcium channel antagonist, the mechanically induced increases in bone formation and adaptation are substantially suppressed [5]. Calcitropic hormones, such as parathyroid hormone (PTH) and activated vitamin D3, also exert their effects through voltage-dependent calcium channels [6, 7].

The voltage-dependent L-type calcium channel is a multi-subunit complex composed of the pore-forming α_1_ subunits along with accessory β, α_2_δ and γ subunits. There are three types of α_1_ subunit: α_1c_, α_1d_, and α_1s_. The α_1c_ subunit is found to be the most abundant in rodent osteoblasts which serve as the primary site for Ca^2+^ influx [8, 9]. Thus far, four types of β subunits have been identified: β_1_, β_2_, β_3_, and β_4_. The β subunit facilitates the proper folding of α-subunit and its transport to the membrane, and is also very important in carrying on signal transduction processes [10, 11]. The mRNA of α_1c_, α_1d_, α_1s_, and all four β subunits can be detected in osteoblast. However, only α_1c_ and β_1–3_ can be detected using western blot analysis [12, 13]. We identified abundant β_3_ expression in Ros 17/2.8 osteoblast cell [13].

The actin cytoskeleton in osteoblast cells supports the assembly of L-type calcium channel complexes and its attachment to the plasma membrane. This is supported by our previous study showing that disruption of actin cytoskeleton resulted in a negative shift in inactivation voltage and a decrease in peak current [13]. The role of filamentous actin (F-actin) in regulating channel activities relies on actin binding proteins, including actin filament severing protein which depolymerizes actin, and actin crosslinking protein which facilitates the binding of actin to the channel. Actin-binding protein actinin cross-links F-actin bundles or networks as scaffolding proteins which played critical role to regulate various ion channels and transporters [14, 15, 16]. Actinin can be grouped into two distinct classes: muscle isoforms (2 and 3) and non-muscle cytoskeletal isoforms (1 and 4), they have different tissue types and expression profiles. In addition to its actin binding property, actinin also serves as a scaffolding protein to connect the cytoskeleton to diverse signaling pathways [17].

Forskolin regulates L-type calcium channel activities by the well recognized phosphorylation modification of the channel proteins through cAMP dependent protein kinase A (PKA) with the help of A-kinase anchoring proteins (AKAPs) [18, 19, 20, 21, 22]. It has also been linked to the regulation of actin reorganization and the phosphorylation of actin itself and actin-binding proteins [23, 24].

Our previous study in osteoblasts identified the regulatory role of the actin cytoskeleton on L-type calcium channel activity [13]. The disruption of actin cytoskeleton alters the kinetic properties of the L-type calcium channel. Here, we report that the L-type calcium channel proteins interacted with scaffolding protein actinin 4 in osteoblast cells, and the calcium channel activities were modulated through the effects of forskolin on actin reorganization and on the functional interaction of calcium channel proteins with actinin 4.

## Materials and Methods

### Cell Culture

Ros 17/2.8 cells were grown in Ham’s F12 medium (Sigma, St. Louis, MO, USA), supplemented with 10% heat-inactivated newly born calf serum (GIBCO BRL, Co.Ltd, USA), 100 U/ml penicillin and 100 U/ml streptomycin. The cells were passaged at 90% confluence by treatment with trypsin-EDTA and seeded in 75-mm^3^ flasks. The cells were cultured in a 5% CO_2_ incubator at 37°C. For patch-clamp experiments, cells were seeded at low density on cover slips.

### Plasmid Constructs

Actinin 4 plasmid constructs were kindly given by Dr. Mark Donowitz (Departments of Medicine/Division of Gastroenterology at the Johns Hopkins University School of Medicine). Various domains of actinin 4 were generated by PCR-based strategy from the actinin 4 cDNA. These domains include ABD (corresponding to amino acids 1–237), R14 (aa 269–724), and EF (aa 742–811). The PCR products were shuttled into pGEX4T-1vector (Amersham Biosciences, Piscataway, NJ, USA) using *Eco*RI and *Xho*I sites introduced by PCR. These were expressed as glutathione *S*-transferase (GST)-tagged fusion proteins in BL-21 cells (Stratagene, La Jolla, CA, USA), and affinity-purified with glutathione-Sepharose as suggested by the manufacturer. The fidelity of PCR products were confirmed by DNA sequencing. HA-β_3_ and α_1c_ plasmids were kindly given by Dr. Sandra E Guggino (Departments of Medicine/Division of Gastroenterology at the Johns Hopkins University School of Medicine).

### Transfection

Ros 17/2.8 cells were plated on 35 mm culture dishes. When the cells reached 50%~60% confluence, the transfection was carried out following the Lipofectamine 2000 (Lifetechnology, Carlsbad, CA, USA) instruction. For each dish, 1 μg plasmid DNA and 2 μL Lipofectamine 2000 were added into 100 μL medium without serum and antibacterial agent. The cells were cultured in a 5% CO_2_ incubator at 37°C. The medium was changed into the normal medium 6 h later, and the cells are used for further experiment 48 ~ 72 h after transfection.

### Whole-cell patch-clamp experiment

The cells were plated at low density onto cover slips coated with Vitrogen (Cohesion, Palo Alto, CA, USA). The perforated patch recording technique was used to measure inward barium currents under voltage clamp conditions. β-escin creates small nonselective pores in the membrane which allows ion flow but large intracellular molecules remain in the cell. The final concentration of β-escin was 50μM in the cytosolic internal solution containing 100 mM KOH, 150 mM HEPES, 20 mM EGTA, 2 mM CaCl_2_, 2 mM MgCl_2_, 10 mM K_2_HPO_4_, buffered to pH 7.4 with KOH. The composition of initial external solution was 140 mM NaCl, 5 mM KCl, 20 mM HEPES, buffered to pH 7.4 with NaOH. After whole-cell currents were established, a solution consisting of 115 mM BaCl_2_ and 20 mM HEPES solution buffered to pH 7.4 with tetraethylammonium hydroxide was added to the initial solution so that the final concentration of barium was 20 mM. Potassium currents were blocked by tetraethylammonium in the external solution.

A pipette was positioned at the surface of a cell, and then gentle suction was applied until a tight seal of about 10 GX was formed. After about 1–3 min the β-escin diffused into the cell membrane under the patch pipette and the capacitance decreased, at which time the experiment was initiated. The cell membrane potential was held at -70 mV; 10-mV step pulses were applied for 200 ms between—30 and +70 mV. Currents were recorded on an EP7 patch-clamp amplifier (List Electronics, Darmstadt, Germany). Pclamp 8 was used for data collection and analysis.

### Staining Actin Stress Fiber

Ros 17/2.8 cells were seeded on chamber slides (Nalge Nun International, Naperville, IL, USA). At 70% confluence, the cells were treated with forskolin at 10, 20, 30 and 50 μM for 24 h. They were then fixed with 4% paraformaldehyde in 0.1 M phosphate buffer (PBS). Following washes with PBS, the cells were permeabilized with 0.1% Triton X-100 for 5 min at room temperature. The cells were blocked with 1% BSA for 30 min at room temperature, and then incubated with Alexa 568 phalloidin (Molecular Probes, Eugene, OR, USA) for 20 min at room temperature. After three washes with PBS, cells were counterstained with DAPI nuclear stain for 5 min and mounted with Vectashield. The changes of actin cytoskeleton after treatment with the different concentrations of forskolin were visualized by confocal microscopy (LSM 410; Carl Zeiss, Oberkochen, Germany). The presence of actin stress fibers was determined by evaluating minimal five samples.

### Surface biotinylation

Ros 17/2.8 cells were plated on 75-mm^3^ flasks. When the cells reached 90–95% confluence, the surface biotinylation assay was carried out [25].The cells were placed on ice and washed three times with ice-cold Dulbecco’s phosphate buffered saline (DPBS–Ca-Mg), and then the cells were incubated with 1 mg/ml NHS-SS-biotin (Pierce, Rockford, IL, USA) in DPBS–Ca-Mg for 30 min at 4°C with gentle shaking. After biotinylation, the cells were rinsed once and washed twice with 100 mM glycine in PBS–Ca-Mg to quench the reaction. After an additional two washes with cold DPBS–Ca-Mg, the cells were then scraped and solubilized with RIPA buffer containing the protease inhibitors as used for protein isolated for the Western blots. The lysates were centrifuged at 6,000 g for 10 min at 4°C. The supernatants were collected, from which 1/20 was taken as an estimate of the input, and the rest was incubated with 200 μL streptavidin–agarose beads (Pierce). To ensure the complete recovery of biotinylated protein, the extraction with streptavidin–agarose beads was performed two times. The first extraction was performed overnight and the second for 2 h. After washing the beads with RIPA buffer five times, 50 μL 2×Laemmli sample buffer (Bio-Rad, Hercules, CA, USA) containing 200 mM DTT was added and incubated at 37°C for 1 h to elute the biotinylated protein. The elution process was also performed two times to ensure complete recovery. After centrifugation at 14,000 g for 2 min, the eluates were collected for western blot analysis using the α_1C_ subunit antibody (ACC 003). The densities of the bands in the western blot were analyzed using Scion Image (Scion Corporation, Frederick, MD, USA).

### Pull-down assay

The pull-down assay was performed using the purified GST-actinin 4 fusion protein to pull down the L-type calcium channel subunits interacted with actinin 4 in Ros 17/2.8 cells, which were then detected by specific antibodies. 3 μg purified GST fusion protein was bound to glutathione beads (20 μL) for 2 h at 4°C with rotation. The beads were washed vigorously with lysis buffer (1% NP40, 0.5% sodium deoxycholate and 0.1% SDS in PBS) for 5 times for 25 min. The washed beads were incubated with 1~2 mg Ros 17/2.8 lysatses for 2 h at 4°C with rotation. The complexes were washed with lysis buffer for 5 min × 5 times and centrifuged. Bound proteins were eluted with Lammelli buffer for 10 min at 70°C to reducing sample buffer and analyzed by western blotting using the corresponding antibodies.

### Immunoprecipitation

The L-type calcium channel β_3_ subunit was transfected into the Ros 17/2.8 cells. The cells were harvest after transfection for 48~72 h and solubilized with RIPA buffer containing the protease inhibitors as used for protein isolated. The protein in the supernatant was quantified with BCA protein assay kit (Pierce, Rockford, IL, USA). The cell lysates (1~2 mg) were incubated with 60 μL Protein A sepharose beads (Amersham Pharmacia, Uppsala, Sweden) at 4°C for 2 h with rotation to preclear. Meanwhile, 2 μg normal rabbit IgG (negative control) or anti-β_3_ subunit antibody (Alomone, Jerusalem, Israel) was incubated with 80 μL Protein A beads for ~2 h at 4°C with rotation. The beads were washed for 5 min × 5 times. Then the precleared cell lysates were incubated with the antibody conjugated beads for 2 h at 4°C with rotation. The beads were washed for 5 min × 5 times and eluted with 8% SDS Lammelli buffer for 10 min at 70°C to reducing sample buffer. The eluted samples were detected by western blot analysis with corresponding antibodies.

### Immunofluorescence Analysis

Ros 17/2.8 cells and MG63 cells were plated on chamber slides (NalgeNunc International, Naperville, IL, USA). When the cells were extended thoroughly, they were washed three times with PBS and fixed with 4% paraformaldehyde in PBS for 25 min. Following three washes with PBS, the cells were permeabilized with 0.1% Triton X-100 for 5 min at room temperature. Then they were blocked with 1% BSA for 1 h at room temperature followed by three washes with PBS. The cells were incubated with anti-actinin 4 antibody (Abcam, Cambridge, MA, USA) and anti-β_3_ subunit antibody (Alomone, Jerusalem, Israel) at 4°C over night and 30 min at room temperature the next day with gently rocking. After washing with PBS for 10 min for three times, the cells were incubated with the secondary antibodies with labeled with Rhodamine or FITC (Jackson Lab., Barharbor, Maine, USA) at room temperature for 2.5 h shield from light. After three washes with PBS likewise, cells were counterstained with DAPI nuclear stain for 5 min and washed once with PBS, and mounted with Vectashield and reserved at 4°C shield from light. The fluorescent signals were visualized by confocal microscopy (LSM 410; Carl Zeiss, Oberkochen, Germany).The co-localization of actinin 4 and L-type calcium channel β_3_ subunit was determined by evaluating at least five samples.

### Stable shRNA mediated repression of actinin 4 in Ros17/2.8 osteoblast cells

Actinin 4 expression in Ros 17/2.8 cells was silenced by shRNA interference. The lentiviral mediated shRNA against actinin 4 system was purchased from Shanghai Sunbio Medical Biotechnology, Shanghai, China. An adopted non-silencing control hRNA sequence (TTCTCCGAACGTGTCACGT) that was not complementary to any human gene was used as a control shRNA. Lenti viruses were prepared in HEK293T cells followed by infecting Ros17/2.8 osteoblast cells. Cells were selected using 0.5 μg/mL puromycin and subjected to western blot analysis to determine the expression level of actinin 4 in the infected cells.

### Statistical analysis

The data are shown as mean ± SE. Statistical significance was assessed using a Student’s paired t test when there were only two groups involved. In the remaining cases the results were analyzed using one-way analysis of variance (ANOVA) followed by Tukey’s multiple comparison; P<0.05 was considered significant. All data analyses were performed using the software SPSS 15.0.

## Results

### Effect of forskolin on actin cytoskeleton in osteoblast Ros 17/2.8 cells

**Fig 1:**
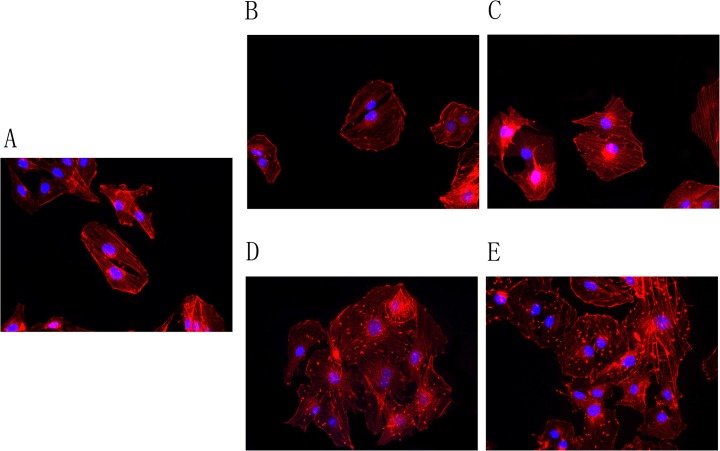
Effect of forskolin on the morphology of actin cytoskeleton in osoteoblast Ros17 / 2.8 cells. Ros17/2.8 cells were treated by forskolin at (A) 0 μM, the control group; (B) 10 μM; (C) 20 μM; (D) 30 μM; and (E) 50 μM for 24 h. The actin fibers were stained with Alexa 568 phalloidin (red) and the nuclei were stained with DAPI (blue). The images show increased cell volume and more protrusions at periphery of the cells as a function of the increased concentration of forskolin (n = 4). Original magnification ×400.

To determine the effect of forskolin (an activator of adenyl cyclase) on actin cytoskeleton in Ros 17/2.8 cells, we treated the Ros 17/2.8 cells with forskolin and stained the cells for actin fibers with Alexa 568 phalloidin. The non-treated Ros 17/2.8 cells were slender, with well-maintained cell integrity and few protrusions at periphery of the cells (Fig 1A). After forskolin treatment, the Ros 17/2.8 cells exhibited increasing cell volume and more protrusions at periphery of the cells, as a function of the forskolin concentration (Fig 1B–1D). The changes of cell morphology in an forskolin dose-dependent manner supports the role of forskolin in inducing actin depolymerization and cytoskeleton reorganization in Ros 17/2.8 cells, which are consistent with the findings seen in many other cell types.

### Effect of forskolin on the expressions of α_1c_ subunit of L-type calcium channel in Ros 17/2.8 cells

**Fig 2:**
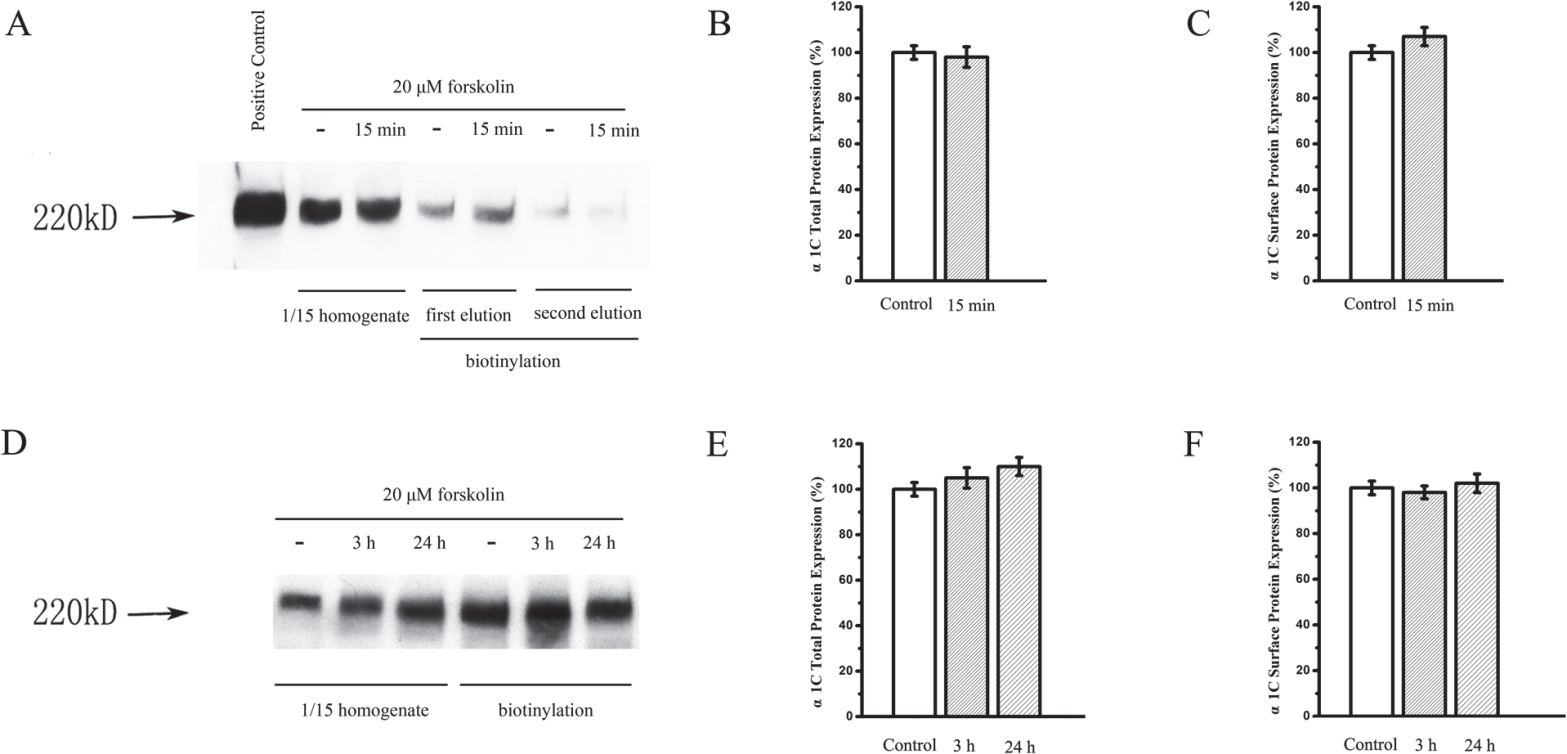
Effect of forskolin on the surface expression of L-type calcium channel α_1c_ subunit inosteoblast Ros17/2.8 cells. (A) Cell surface expression of L-type calcium channel α_**1c**_ subunit of Ros17/2.8 cells is shown after treatment of 20 μM forskolin for 15 min. The quantitative data show the total (B) and surface (C) α_**1c**_ protein levels. There is no significant difference between these groups (n = 3, P>0.05). (D) Cell surface expression of L-type calcium channel α_**1c**_ subunit of Ros17 /2.8 cells is shown after treatment of 20 μM forskolin for 3 h and 24 h. The quantitative datas show the total (E) and surface (F) α_**1c**_ protein levels. There is also no difference between these groups (n = 4, P>0.05).

We tested the influence of forskolin on the expressions of α_1c_ subunit in Ros 17/2.8 cells treated with either vehicle or forskolin to further explore the underlying mechanism of forskolin on the L-type calcium channel. This functional expression of the α_1c_ subunit was analyzed by the cell-surface biotinylation experiments to determine the amount of α_1c_ membrane expression of L-type calcium channel in Ros 17/2.8 cells. The expressions of total α_1c_ and membrane α_1c_ of the L-type calcium channel in Ros 17/2.8 cells, as shown in Fig 2A, were not significantly different between the non-treated control and the 20 μM forskolin treated group for the short time of 15 min (n = 3, p>0.05). The results indicated that forskolin had no effect on the surface expression of α_1c_ subunit for the short period of time. When the forskolin treatment times were extended to 3 h and 24 h, there was still no significant change of surface expression of α_1c_ subunit (Fig 2D, n = 4), even though the Ros 17/2.8 cells started to exhibit change in cell shape at 3 h and there was a trend of increase of the total expression of α_1c_ at 24 h.

### Effect of forskolin on the interaction of L-type calcium channel α_1c_ subunit with actin binding protein actinin 4

**Fig 3:**
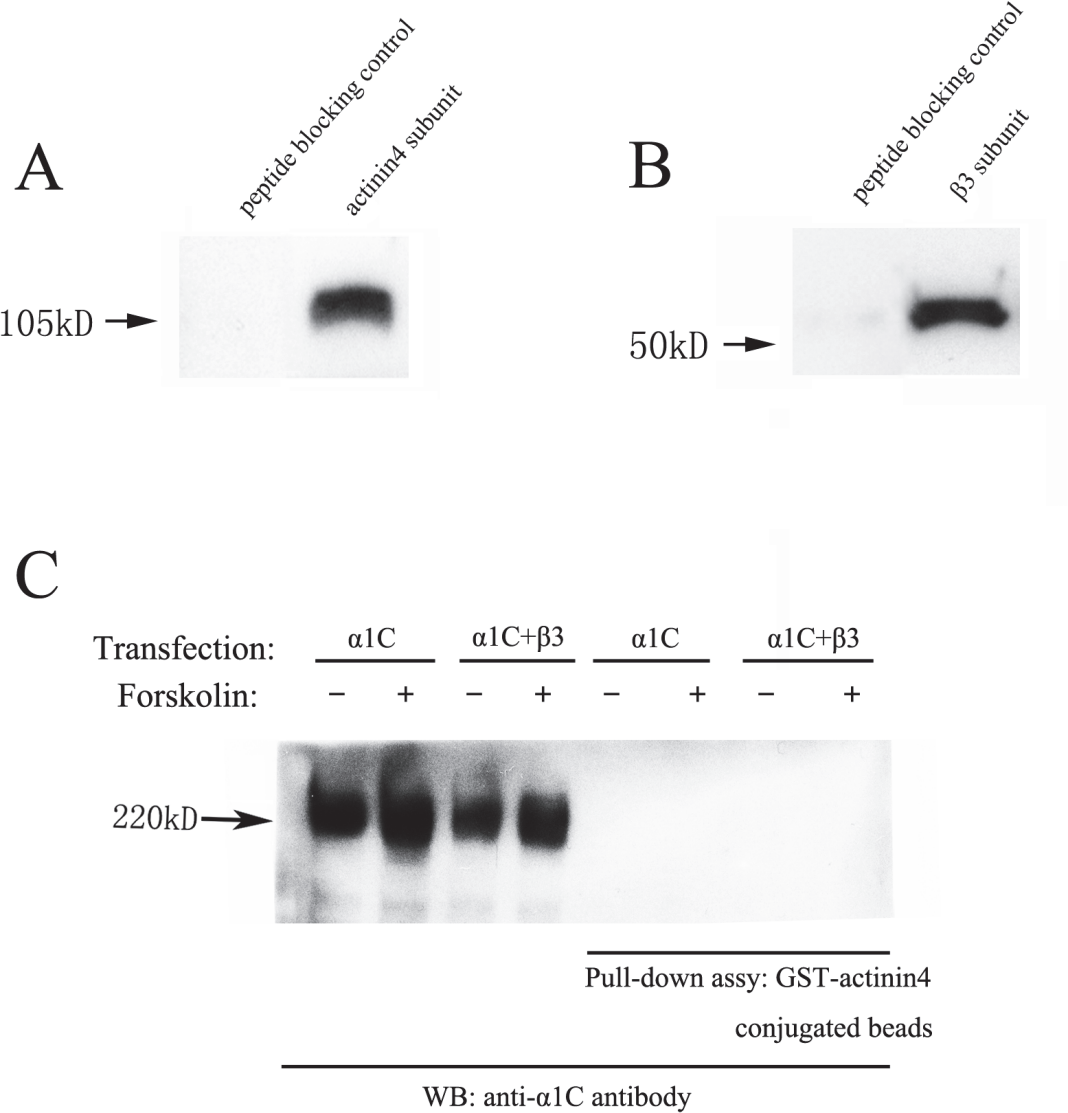
The interaction of actinin 4 and L-type calcium channel α_1c_ subunit with 20 μM forskolin for 24 h. Ros17/2.8 cells were transfected with either L-type calcium channel α_**1c**_ subunit alone or co-transfected with L-type calcium channel α_**1c**_ subunit and β_**3**_ subunit. The cell lysates were pulled down with GST-actinin 4 conjugated beads and then were subjected to western blot analysis, using anti-α_**1c**_ antibody. (A) The expression of actinin 4 in Ros17/2.8 cells. (B) The expression of β_**3**_ subunit in Ros17/2.8 cells. (C) The interaction of actinin 4 and L-type calcium channel α_**1c**_ subunit. The results showed that actinin 4 did not interact with L-type calcium channel α_**1c**_ subunit, and forskolin did not significantly affect the interaction of actinin 4 with α_**1c**_ subunit (n = 3).

As an actin cross-linking protein, actinin 4 is important in maintaining the connection between actin and L-type calcium channel. Western blot analysis showed that actinin 4 and the L-type calcium channel β_3_ subunit were both expressed in Ros17/2.8 cells (Fig 3A and 3B). To assess the interactions of these proteins in Ros 17/2.8 cells, pull-down assay and western blot analysis were used to determine the interaction between these proteins. To increase the signal amplitude of the pull-down assay, the plasmids of α_1c_ and β_3_ subunits were transfected individually or co-transfected into Ros 17/2.8 cells. Western blot analysis of these pulled-down proteins using anti-α_1c_ subunit antibody, did not reveal the interaction between actinin 4 and L-type calcium channel α_1c_ subunit (Fig 3C). Treatment with forskolin did not promote the interaction (n = 3). We also examined the dystrophin, another actin binding protein, and the results showed no interaction between dystrophin and α_1c_ subunit (data not shown).

### Effect of forskolin on the interaction of L-type calcium channel β_3_ subunit with actin binding protein actinin 4

**Fig 4:**
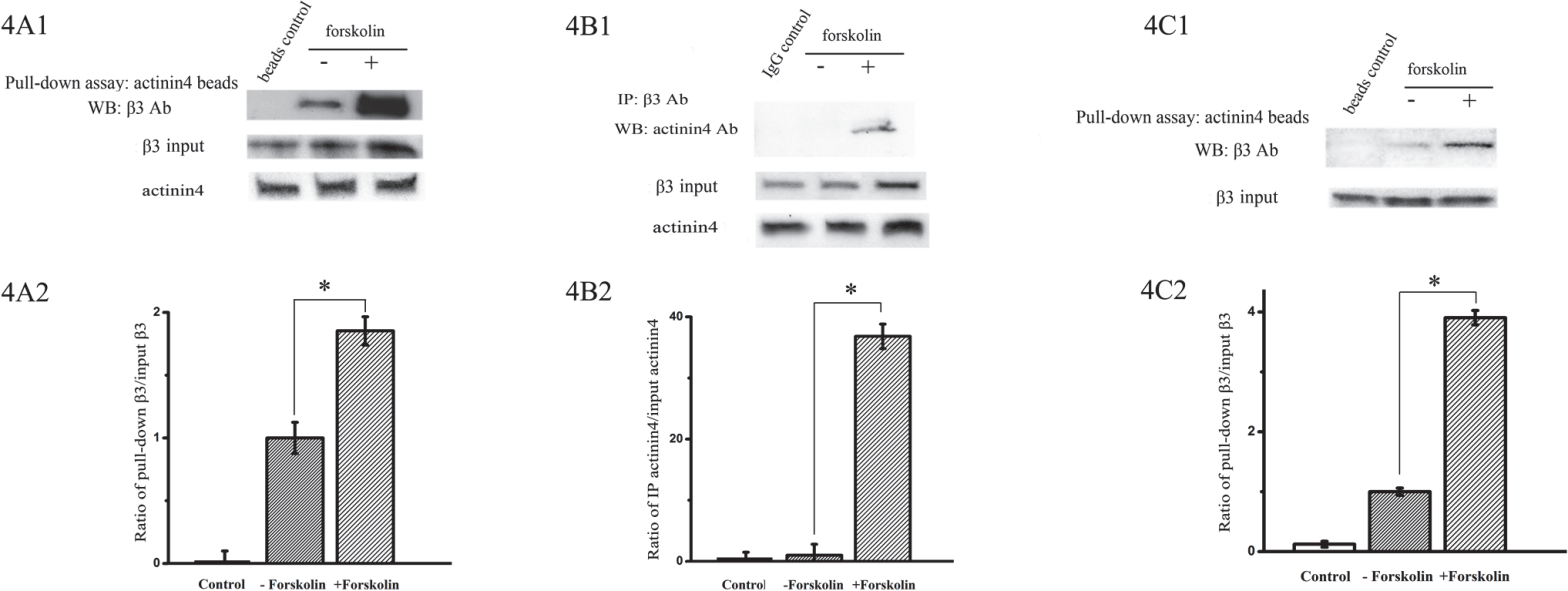
The interaction of actinin 4 and L-type calcium channel β_3_ subunit with 20 μM forskolin treatment for 24 h in osteoblast Ros17/2.8 cells. Cells were transfected with L-type calcium channel β_**3**_ subunit. (A1) Sample was pulled down with GST-actinin 4 conjugated beads, and immunoblotted with anti-β_**3**_ subunit antibody. (A2) The quantitative ratio of pull-down β_**3**_ over the input β_**3**_ subunit (P<0.05, n = 6). (B1) Sample was immunoprecipitated with anti-β_**3**_ subunit antibody, and immunoblotted with anti-actinin 4 antibody. (B2) The quantitative ratio of IP actinin 4 over the input actinin 4 (P<0.05, n = 3). (C1) The cell lysates were treated with forskolin *in vitro* and then pulled down with GST-actinin 4 conjugated beads. Anti-β_**3**_ subunit antibody was used to detect the protein by western blot analysis. (C2) The quantitative ratio of pull-down β_**3**_ over the input β_**3**_ subunit *in vitro* (P<0.05, n = 3).

We then performed co-immunoprecipitation and pull-down assay to examine the interaction of actinin 4 and β_3_ subunit of L-type calcium channel in Ros 17/2.8 cells with transfected β_3_ subunit plasmid. Fig 4A showed that after GST pull-down with GST-actinin 4 conjugated beads, β_3_ subunit was detected at a much higher quantity in forskolin-treated Ros17/2.8 cells compared to that in non-treated Ros17/2.8 cells. Since the total expression of β_3_ subunit was also increased in the forskolin treated group, we did the quantitative analysis of ratio of pull-down β_3_ over the input β_3_ subunit. The results showed that the ratio is significantly increased in forskolin-treated cells(P<0.05, n = 6). Reciprocally, after immunoprecipitation with anti-β_3_ antibody, actinin 4 was detected only in forskolin treated Ros17/2.8 cells (Fig 4B) (n = 3). To further verify this finding, we performed the pull-down assay with forskolin *in vitro* in cell lysates so that the same amount of input β_3_ subunit was used. When the cell lysates were treated with 20 μM forskolin for 24 h *in vitro* and incubated with GST-actinin 4 conjugated beads, β_3_ subunit was also detected at higher quantity than the non-forskolin treatment (Fig 4C, n = 3). These results suggested that the actin binding protein actinin 4 interacts with β_3_ subunit and forskolin enhances their interaction. The dystrophin was not found to interact with β_3_ subunit, a similar finding with α_1c_ subunit (data not shown).

### Effect of H89 on the interaction of actinin 4 and β_3_ subunit of L-type calcium channel in osteoblast Ros 17/2.8 cells

**Fig 5:**
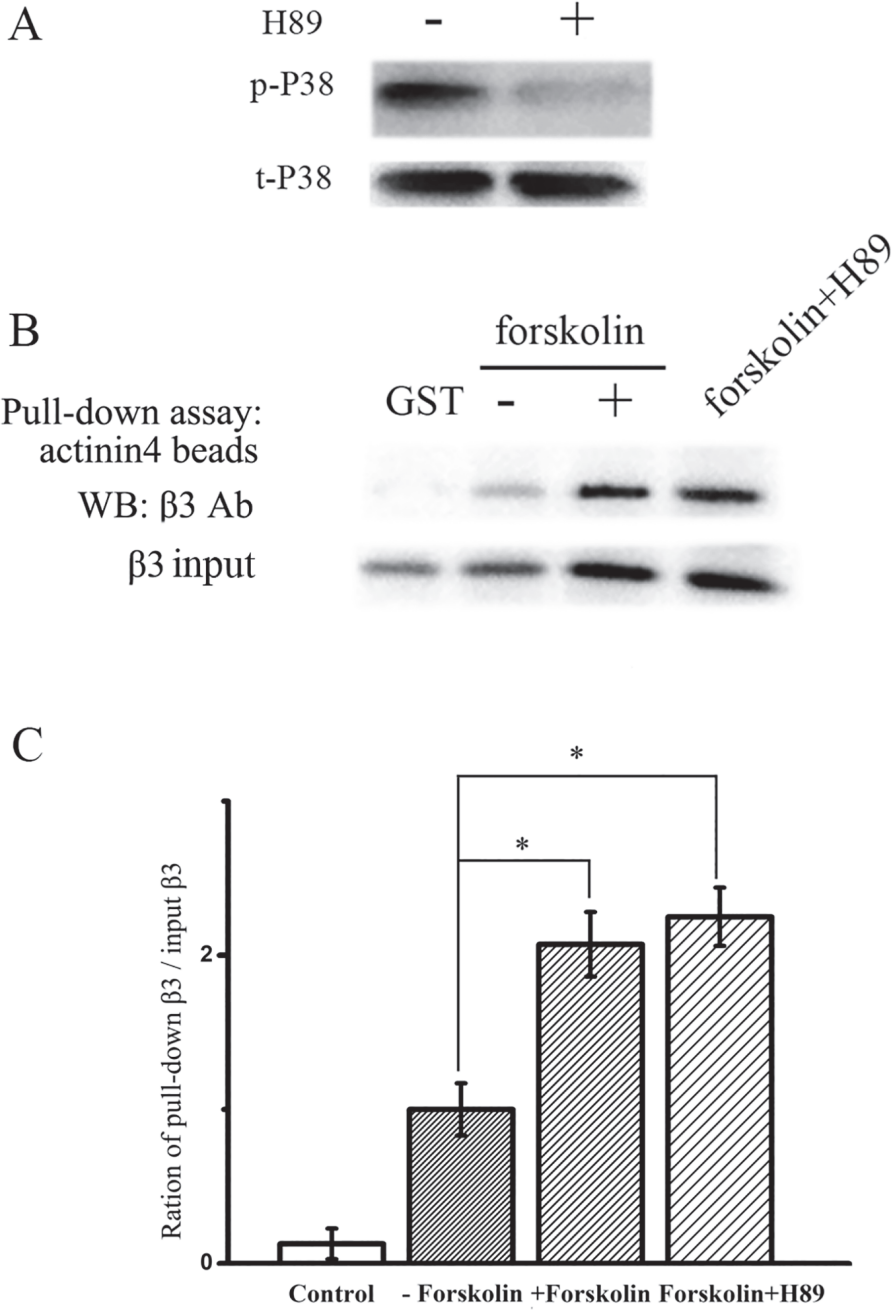
Effect of H89 on the interaction between actinin 4 and L-type calcium channel β_3_ subunit after treatment with 20 μM forskolin for 24 h in osteoblast Ros17 / 2.8 cells. Cells were transfected with L-type calcium channel β_**3**_ subunit. The interaction between L-type calcium channel β_**3**_ subunit and actinin 4 is increased in either forskolin alone treatment group or forskolin and H89 co-treatment group compared with the control group. (A) The effect of H89 on the expression of phosphorylated P38 and total P38. (B) The effects of H89 and forskolin on the interaction between actinin 4 and β_**3**_ subunit. (C) The quantitative ratio of pull-down β_**3**_ over the input β_**3**_ subunit (P<0.05, n = 3).

The above experimental results demonstrated that the actin-binding protein actinin 4 interacts with L-type calcium channel β_3_ subunit, and that forskolin enhances their interaction. To further determine the incorporated effect of forskolin mediated phosphorylation of L-type calcium channel on this interaction, Ros 17/2.8 cells were treated for 24 h in the presence of 5 μMH89, a specific inhibitor of PKA, along with 20 μM forskolin. P38 is the downstream of forskolin signaling [26]. Thus, we used the p38 as a marker to assess the effects of H89. Fig 5A showed that H89 inhibited the expression of phosphorylated P38 and had no effect on the total P38, suggesting H89 could inhibit PKA mediated phosphorylation effectively. As shown in Fig 5B, compared to the non-treated control, the interaction between the β_3_ subunit of L-type calcium channel and actinin 4 was significantly increased in Ros 17/2.8 cells treated with either forskolin alone or co-treated with forskolin and H89. However, the interaction between the β_3_ subunit and actinin 4 in the forskolin treatment alone group was not different from that in the forskolin-H89 co-treatment group (n = 3), indicating that the interaction of the β_3_ subunit of L-type calcium channel with actinin 4 is independent of PKA mediated phosphorylation.

### Effect of forskolin on the co-localization of actinin 4 with β_3_ subunit of L-type calcium channel

**Fig 6:**
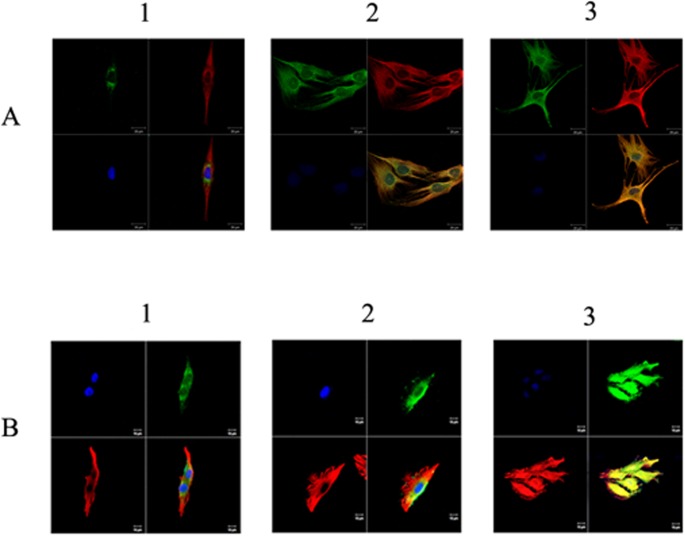
Eeffect of forskolin on cell morphology and co-localization between actinin 4 (red) and L-type calcium channel β_3_ subunit (green) in osteoblast Ros 17/2.8 cells and MG 63 cells. Nuclei stained by DAPI (blue). (A1) No forskolin treatment in Ros 17/2.8 cells (n = 6); (A2) Treatment with 20 μM forskolin for 3 h in Ros17/2.8 cells (n = 6); and (A3) Treatment with 20 μM forskolin for 24 h in Ros17/2.8 cells (n = 6). (B1) Non-treated control group in MG 63 cells (n = 6); (B2) Treatment with Forskolin + IBMX for 40 min in MG 63 cells (n = 6); (B3) Treatment with Forskolin + IBMX for 3 h in MG 63 cells (n = 6).

Fig 6 shows the co-localization between actinin 4 and β_3_ subunit after immunofluorescence dual labeling staining for actinin 4 and β_3_ subunit in Ros17/2.8 cells treated with 20 μM forskolin for different time periods. In non-treated group, cells appear slender, with well-maintained cell integrity and few protrusions at periphery of the cells. Actinin 4 is distributed in the cytoplasm evenly, and the β_3_ subunit is concentrated around the nucleus (Fig 6A1). No co-localization of actinin 4 with β_3_ subunit is observed. After treatment with 20 μM forskolin for 3 h, the cell volume appears to be increased with more protrusions at the periphery of the cells, and the β_3_ subunit of L-type calcium channels appears to be in the cytoplasm instead of the periphery of nucleus. The distribution patterns of β_3_ subunit are similar to the actinin 4 (Fig 6A2). When treatment with 20 μM forskolin was extended to 24 h, the cells exhibited further expanded volume and even more protrusions. The fluorescent signals of actinin 4 and β_3_ subunit are superimposed completely in yellow, indicating a co-localization between these two proteins (Fig 6A3). These results suggested that forskolin induces the increase of the cell volume and cytoplasm protrusions in Ros 17/2.8 cells, consistent with our findings on forskolin’s effect on actin cytoskeleton as shown in Fig 1. They also indicate that forskolin promotes the transport of β_3_ subunit from the periphery of nuclear to cytoplasm and the interaction of β_3_ subunit with actinin 4, since the co-localization of actinin 4 with β_3_ subunit increases as a function of the forskolin treatment time (n = 5). These findings are in accord with the observed effect of forskolin on interaction between actinin 4 and β_3_ subunit as shown by co-immunoprecipitation studies (Fig 4).

In addition, we performed similar experiments in human osteoblast-like cells, MG63 cells. Cells were subjected to combined treatment of forskolin and IBMX (a nonspecific inhibitor of phosphodiesterase) to increase cAMP formation and decrease the degradation of cAMP. After treatment with 10 μM forskolin and 100 μM IBMX for 40 min, the MG63 cells exhibit change of cell shape with increased cell volume compared to the cells in the non-treated control group (Fig 6B1). Again, the β_3_ subunit of L-type calcium channels appears to be translocated to the cytoplasm from the periphery of nucleus (Fig 6B2). When the same combined treatment was extended to 3h, the β3 subunit’s distribution in the cytoplasm appears to be homogeneous, and the fluorescence of the β3 subunit (green) is superimposed with that of actinin 4 (red) throughout the cell (Fig 6B3) (n = 5). These results from MG63 cells are consistent with our above-mentioned findings that forskolin induces the increase of the cell volume and cytoplasm protrusions in a time dependent manner and enhances the co-localization of actinin 4 with the β3 subunit in Ros 17/2.8 cells.

### Mapping of interaction site of actin binding protein actinin 4 and L-type calcium channel β_3_ subunit in osteoblast Ros 17/2.8 cells

**Fig 7:**
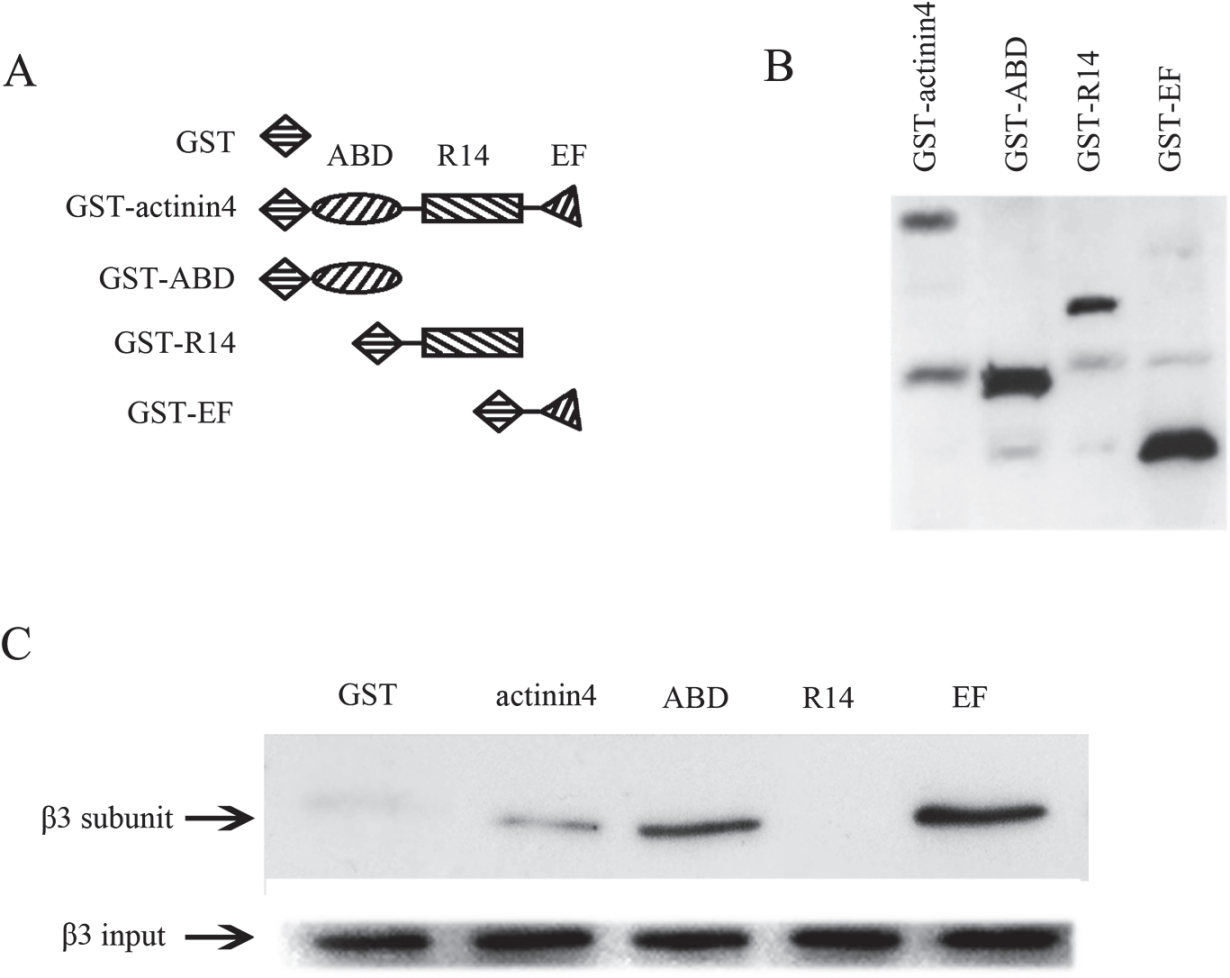
The interaction between actinin 4 and L-type calcium channel β_3_ subunit via the actin-binding domain (ABD) and EF-hand domains (EF), not in the spectrin-repeat domains (R14). (A) The picture shows some representative domains of actinin 4, including ABD (actin-binding domain), R14 (spectrin repeat domain 1~4) and EF (EF-hand motif). (B) A representative western blot of GST fused actinin 4 fragments. (C) The pull-down assay results indicate that L-type calcium channel β_**3**_ subunit interacts with actinin 4 at its ABD and EF domain (n = 3).

To further demonstrate our understanding on the interaction of actinin 4 with L-type calcium channel β_3_ subunit, we explored the binding sites in actinin 4. Actinin 4 has an actin-binding domain, four spectrin-like repeats, and two EF-hand domains [15, 27]. Various constructs of actinin 4 were generated as GST fusion proteins. The results showed that β_3_ subunit binds to the actin-binding domain (ABD) and EF-hand domains of actinin 4, but not to the spectrin-repeat domains (R14) (Fig 7C, n = 3). These results revealed that the interaction of actinin 4 with L-type calcium channel β_3_ subunit is mediated by the actin-binding domain and EF-hand domains of actinin 4. Combined with previous findings, these data demonstrated that actinin 4 interacts with β_3_ subunit (Fig 4).

### Functional interaction of actinin 4 and the L-type calcium channel in osteoblast Ros 17/2.8 cells

**Fig 8:**
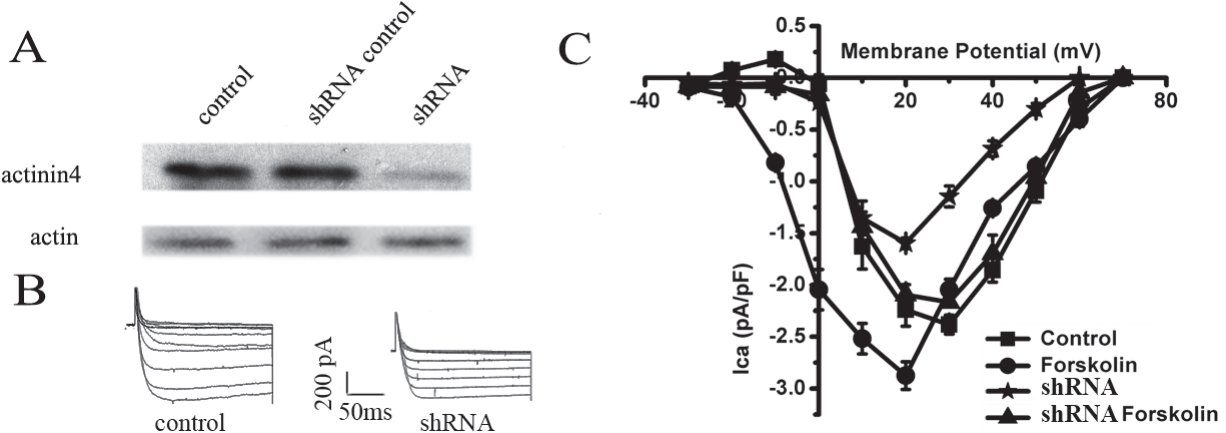
Functional effect of actinin 4 on L-type calcium channel in osteoblast Ros 17/2.8 cells. (A) shRNA interference silenced the expression of actinin 4 in Ros 17/2.8 cells, and the effect was not exhibited on actin. (B) The representative traces of L-type calcium channel in control and actinin 4 shRNA group. (C) Current-voltage relationship of L-type calcium channel. The actinin 4 shRNA decreased L-type calcium channel currents in control (P<0.05, n = 6) and forskolin treated group (P<0.05, n = 6).

To further determine the functional significance of the interaction between actinin 4 and Ca channel, the L-type calcium channel currents (I_*Ca*_) were recorded using the whole-cell patch clamp technique in Ros17/2.8 cells. As expected, forskolin considerably increases I_*Ca*_ (Fig 8C). The peak current density was significantly increased from 2.38±0.21 pA/pF (control group, n = 6) to 2.87±0.31 pA/pF (forskolin group, n = 6) (*P*<0.05). When actinin 4 expression was specifically knocked down by shRNA (Fig 8A), the current of L-type calcium channel was significantly decreased in both of the control and forskolin treated group (Fig 8B), the current density was decreased to 1.59±0.06pA/pF (*P*<0.05, n = 6) and 2.16±0.11pA/pF (*P*<0.05, n = 6) respectively (Fig 8C). These results suggested that actinin 4 is critical for the L-type calcium channel function in Ros 17/2.8 cells and the elimination of actinin 4 leads to reduction of the L-type calcium channel’s activities.

## Discussion

The Ca^2+^ level in osteoblast cells is important to osteoblast cellular activities and hence to bone density [20]. Our previous work has shown that the depolymerization of the actin cytoskeleton in Ros 17/2.8 cells with cytochalasin D (CD) significantly reduced the L-type calcium channel current and shifted the voltage at half-maximal inactivation to a more negative potential. We also found that CD did not change activation kinetics or surface expression of the pore-forming α_1c_ subunit. The net result of actin depolymerization would close the channel at more negative potentials, thereby reducing inward calcium movement. Our work [13] and the others’ have linked the assembly and disassembly of F-actin to the L-type calcium channel kinetics as well as the trafficking of the channel.

Our present studies were focused on the changes of the actin cytoskeleton and the L-type calcium channel complexes in osteoblasts induced by forskolin, and how the changes affect the channel activities. Forskolin activates PKA which in turn phosphorylates the subunits of the L-type calcium channel and results in enhancement of its activity and the increase of I_*Ca*_. Our data have shown that treatment of Ros 17/2.8 cells with the adenyl cyclase activator forskolin led to reorganization of the actin cytoskeleton marked by aggregation and reassembling of actin fibers, and resulted in the changes of cell morphology. The change of actin cytoskeleton caused by forskolin is correlated with the effect of forskolin on the I_*Ca*._

However, the reorganization of actin cytoskeleton caused by forskolin has no effect on the membrane expression of α_1c_ subunits of L-type calcium channel in Ros 17/2.8 cells, and we did not see the interaction of actinin 4 and L-type calcium channel α_1c_ subunit. We originally performed the experiments only in lysates with α_1c_ transfection, and did not see any interaction with actinin4. Since β_3_ subunit is able to facilitate the trafficking of α_1c_ subunit, we wonder whether the facilitated α_1c_ subunit is able to interact with actinin4 in the pull-down assay. Thus, we performed the experiments in cells co-transfected with α_1c_ and β_3_ subunits. We did not see the interaction in our solubilizaion conditions either.In the meantime, forskolin treatment caused the translocation of the β_3_ subunit of the L-type calcium channel to the proximity of plasma membrane and the enhanced interaction of the β_3_ subunits with actin binding protein actinin 4, as shown by co-localization images and co-immunoprecipitation technique. So even though we could not rule out interaction between actinin 4 and α_1c_, β_3_ subunits may have stronger interaction with actinin4. As part of the L-type calcium channel, the intracellular cytoplasmic β_3_ subunit interacts with and increases the functional expression of the α_1c_ subunits at the plasma membrane [28, 29], and also facilitates the appropriate folding and membrane localization of L-type calcium channel by blocking an endoplasmic reticulum (ER) retention signal in the I-II intracellular loop of the α_1c_ subunit [10, 11]. β_3_ subunit has structural similarity to the membrane associated guanylate kinase proteins, which function as scaffolds to cluster ion channels and as receptors to transduce intracellular signaling pathways [30, 31, 32]. The membrane-associated guanylate kinase-like properties of β_3_ subunits modulate α_1c_ subunit function [33, 34] and may influence intracellular signal transduction through various protein interactions [35]. Therefore, effect of forskolin on β_3_ subunits in Ros 17/2.8 cells contributes to the enhanced L-type calcium channel activity.

Furthermore, our data demonstrated the novel interaction of L-type calcium channel β3 subunit with actinin 4 in Ros 17/2.8 cells as well as in MG 63 cells. As a scaffolding protein, actinin 4 could facilitate the trafficking and transporting of the L-type calcium channel when it is interacting with the β_3_, as shown in our cell morphological studies. On the other hand, as a common component of the submembranous cytoskeleton, actinin 4 could further support the stability of the L-type calcium channel through its interaction with the β_3_ subunit in addition to its interaction with actin.

Previous study showed that actinin 1 stabilizes α_1c_ subunits at the plasma membrane and the disruption of actinin function reduces the surface localization of L-type calcium channel α_1c_ subunits in HEK 293 and neuronal cultures [36], and actinin 2 interacts with α_1c_ subunits in cardiac myocytes [37]. Another scaffolding protein ahnak was found to associate with the L-type calcium channel via the accessory β_2_ subunit, but not with the β_1_ subunit, in osteoblastic MC3T3-E1 cells, cardiac tissues and T cells [38, 39]. But the regulatory role of actin cytoskeleton in excitable cells may differ from its role in the non excitable cells. For example, in cultured adult rat ventricular myocytes, actin depolymerization alters the trafficking of voltage-dependent L-type calcium channels from the perinuclear region to transverse tubules, where they are normally located and provide the trigger for calcium release [40], whereas actin depolymerization in osteoblast cells affects L-type calcium channel activity independent of protein trafficking [13]. In our study, the loss of function test using shRNA and whole-cell patch clamp technique indicate that actinin 4 is essential for the L-type calcium channel function in Ros 17/2.8 cells. The revelation of the interaction between the β_3_ subunit and actinin 4 in our data, further ascertain the tie of actin cytoskeleton to the L-type calcium channel, where the L-type calcium channel structural and stability are supported by the actin cytoskeleton and the integration of multifaceted interactions of all components of the channel assembly.

Adenylyl cyclase and cAMP have been shown to have the ability to directly affect actin cytoskeleton. Adenylyl cyclase can increase the cortical actin [41]. cAMP plays a role in maintaining the structure integrity and remodeling of the actin cytoskeleton. By doing so, cAMP also contributes to the regulation of adenylyl cyclase since adenylyl cyclase mobility inside the cell is dependent on the dynamic assembly and disassembly of the actin cytoskeleton. In our studies, the forskolin activation of adenylyl cyclase—cAMP cascade mediated increase of the I_*Ca*_ in Ros 17/2.8 cells is correlated to the reorganization of the actin cytoskeleton, the translocation and the interaction of the β_3_ subunits of the L-type calcium channel with actin binding protein actinin 4. The PKA inhibitor H89 did not cause interaction changes induced by forskolin in Ros 17/2.8 cells. We speculate that the observed effects of forskolin on the I_*Ca*_, at least in part, in Ros 17/2.8 cells may be mediated by actin reorganization effects of adenylyl cyclase and cAMP, besides and beyond the PKA mediated phosphorylation of the subunits of the channel.

Our study showed that the binding of the L-type calcium channel β_3_ subunit to actinin 4 was mapped to the ABD on the N-terminus and the EF-hand domains on the C-terminus of actinin 4, but not to its spectrin-repeat domains in the middle region. The data not only confirm the interaction between actinin 4 and the β_3_ subunits, but also coincides with the structural and functional characteristic of actinin where ABD is in close proximity to actin and EF-hand domains may potentially influence the actin-binding activities and regulate calcium binding, while the spectrin-repeat domains serve solely as structural spacers to separate the C-terminus from the N-terminus [42].

In summary, our study further demonstrated the influence of the actin cytoskeleton on the L-type calcium channel activity in osteoblasts. To the best of our knowledge, this is also the first study to identify the interaction of the β_3_ subunits of the L-type calcium channel with actinin 4 in osteoblast cells. This study also revealed a new aspect of the mechanism by which the adenylyl cyclase—cAMP cascade regulates the L-type calcium channel, besides the PKA mediated phosphorylation of the subunits of the channel. The interconnection and dynamics of adenylyl cyclase, cAMP, PKA, actin cytoskeleton and the channel proteins are essential but complex. Further dissection of the intricate mechanisms will enhance our understanding of Ca^2+^-dependent cellular processes in bone.
